# Private Lunatic Asylums in Ireland
*From the "Seventh Report of the Inspectors of Irish Asylums for the Insane."


**Published:** 1855-10-01

**Authors:** 


### PRIVATE LUNATIC ASYLUMS IN IRELAND.*

The number of private asylums in operation at present is the same as mentioned in our last Report; the lunatics, however, have increased by thirty-six, a circumstance corroborative of a fact which our experience in public institutions fully bears out, that insanity is not on the decline in this country, proportionate with the reduction in the general population.

The improvements observable from year to year, since the passing of the Act, afford the strongest proof of the foresight and wisdom of the Legislature in thus particularizing the duties of the inspectors, and giving them the authority of an Act of Parliament to enter into details, and the mode of conducting these establishments, as also to correct any abuses or irregularities they may find to exist in them.

At the date of our First Annual Report, which was made in the year 1843, twelve months after the passing of the 5th and 6th Victoria, the patients confined in private licensed asylums amounted to 373, of whom 161 were males, and 212 females. From that period up to the present they have been annually increasing, there being under treatment on the 1st January, 1855, 459, viz., 252 males, and 207 females; showing an increase of eighty-six lunatics, or twenty-three per cent. on the number under treatment a year after the Act had come into operation.

The most remarkable feature in the above statement is the variation in the proportion of the sexes, the females having exceeded the males in 1843 by fifty-one, and the males exceeding the females in 1854 by forty-five. This

* From the "Seventh Report of the Inspectors of Irish Asylums for the Insane."

variation we find, by reference to the registry kept in our office, is owing to the circumstance that immediately after the passing of the Act, a far greater number of aged females, labouring under chronic lunacy, than of males, were sent to the various private asylums, their friends being desirous, when an opportunity occurred, to place them in establishments where more comfort and attention could be secured to them than in their own houses.

In the course of ten years, as might be reasonably expected, many of these parties died, and hence the present comparative diminution in the numbers of that sex.

Of the 459 patients under treatment at the date of our returns, 132 are set down as curable, and 281, exclusive of forty-six idiots and epileptics, as probably incurable. Of those, however, designated in their certificates of admission as "probably incurable," we have found by experience, from the improved system of treatment now adopted—mildness and judicious management (with constant care and vigilance on the part of the attendants) having superseded mechanical restraint and a less solicitous regard to the feelings and comforts of these afflicted persons—that many become so far relieved as to be enabled to return to their families, and although with impaired understandings, yet capable, to a certain extent, of enjoying the pleasures of their domestic circles, a degree of recovery that scarcely ever was attained under the old system by the class of lunatics in question.

The idiotic and epileptic patients bear but a small proportion to the aggregate numbers in private asylums, the former being only thirty-six, viz., twenty-two males, and fourteen females; the latter ten, of whom one only is a female.

Having investigated into the history of lunacy as regards certain families, from our personal knowledge of the members affected by it in asylums, we would refer to continued intermarriages, and direct hereditary predisposition, no inconsiderable amount of the cases that have come under our observation, the malady frequently developing itself in the third and fourth generation, and, what may appear extraordinary, leaving the second unaffected.

Generally speaking, when such predisposition exists amongst the immediate or exciting causes, whether physical or moral, none will be found more prevalent than intemperance and dissipation; so intimately, indeed, in many cases, is a love of drinking associated with insanity, that it often becomes most difficult to decide whether it be a symptom or merely a result of the disorder. The official management of patients labouring under depraved moral affections is attended with difficulty, and needs much discretion on our part, from the plausibility of the arguments they adduce when under restraint, and the injustice of which they complain at being unnecessarily deprived of liberty, or more truly speaking, of the opportunity to indulge in a reckless dissipation. A majority of the relapsed cases sent to private asylums, and composed of the classes in question, is for the most part carried off by repeated attacks of paralysis.

Of those under treatment on the 1st of January, nearly one half—223 patients—were of mature age, or from forty to sixty years; 152 from twenty to forty; and only five persons under twenty; the remainder being all advanced in life, or over sixty. With reference to this return, it may be stated that early in life, and more particularly when curative treatment is had recourse to on the first appearance of the malady, the mental faculties are more susceptible, and yield more easily to moral and medical influences; so that it frequently happens, under these favourable circumstances, that a few months are sufficient, if not to establish a cure, to afford, at least, well-founded anticipations of ultimate recovery. On the other hand, when the friends of lunatics, from mistaken kindness and motives of delicacy neglect for a long time to place them in an asylum, or to pursue a judicious line of management in their regard, or, above all, when insanity is developed late in life, the probability of recovery becomes slight indeed; the mental, like the corporeal faculties, possessing in advanced age less recuperative powers. Thus the disproportion just referred to may be fairly accounted for.

According to a synopsis of the general social condition of patients, it appeared that the unmarried predominated to a very large extent, there being 354 single or unmarried, and 105 married—majority of unmarried, 249; in the two preceding years it was 253 and 241 respectively, thus showing a uniformity of scale on this head.

With regard to occupations, professions, &c., there is no material alteration, on the whole, since the date of our last public Report in 1853.

It may be worthy of mention, however, in reference to particular professions, that the number of insane persons belonging to the army and navy is about the same now as in 1843; being in 1843—army, 23; navy, 3. In 1854—army, 25; navy, 3; while the number belonging to the church has increased over threefold, viz., from 6 to 19; to the law, twofold—from 9 to 18 (being an average of 1 insane person in every 220 of these two professions, respectively, that of the general population being estimated at about 1 in every 750); of the medical profession, during the same period, from 4 to 6; students of all classes, 6 to 15. Those under the heading "No Occupation" are mainly comprised of females and persons of independent fortune.

During the two years there were 94 patients discharged cured, which reaches 34 per cent. on the admissions; relieved, 55; incurable, 13; died, 45.

Among the deaths, we regret to report the occurrence of one case by suicide, that of an unmarried lady, Miss J———, aged 43, who was admitted into the asylum on the 31st May, labouring under a violent attack of mania. From the evidence given on the inquest, held by the coroner for the county, it appeared that injunctions had been given to the servant in whose charge the lunatic was placed, not to leave her for a moment until she was relieved by some other nurse or attendant. Four days after admission, she having, as she stated in her evidence, other duties to perform, left the lunatic alone, trusting to a strait waistcoat which she put on (without the knowledge of the superintendent) for security. Returning shortly after, she found Miss J——— suspended from the rail of her bed by the cord or lace of the strait waistcoat, of which she had, by some means not accounted for, managed to divest herself. Life at the time was quite extinct. A minute inquiry took place at the inquest, in presence of the brothers of the deceased lady, who felt satisfied that no blame lay with the proprietor; as, had the attendant strictly adhered to the instructions which she admitted had been issued, the unfortunate occurrence could not have taken place. A verdict in accordance with the facts was found by the jury.

Under all the circumstances connected with the foregoing accident, the only course that presented itself, and which was immediately acted upon by directions from this office, was to dismiss the attendant, and impress upon the others, from the example before them, the great responsibility under which they lie, to pay implicit obedience to the instructions they receive, and the serious consequences that may, at any moment, follow a neglect of duty.

Having occasion to animadvert, in the year 1853, on the state of a private asylum, we recommended the magistrates at quarter sessions in the October of that year to give a conditional licence only. At the last October sessions, one of the inspectors attended at the request of the justices, when the chairman intimated to the various proprietors their determination not to grant a licence for any private asylum in future, without the full approbation of the inspectors as to its management in every detail.

The licences, orders for admission, and medical certificates, notices of discharges, and deaths, &c., with two exceptions, have been generally correct, save in some minor points, not necessary to notice in a report to your Excellency, but which points we have not omitted to observe upon to the parties themselves—a strict adherence both to the spirit and letter of the Act of Parliament being required by us.

The first exception occurred in an establishment in the County Limerick, the proprietor having failed to apply for a renewal of his licence. We felt it our duty immediately to notify the fact to the Clerk of the Peace, for the information of the magistrates at quarter sessions: subsequently, however, being assured that the omission was not intended as an evasion of the law, and on the party undertaking to lodge an application for a fresh licence, to be taken into consideration at the quarter sessions next ensuing, and paying the full amount of fees for which he was liable in the first instance, we did not deem it necessary to interfere further.

The second case was that of a medical gentleman, under whose care we discovered three idiotic patients. Having satisfied us that he acted from ignorance of the law in receiving them without due authority, we forebore to institute those legal proceedings which otherwise it would have been our duty to follow up. Finding his house, however, on inspection, to be well suited for the reception of ten patients, we directed him to take out the necessary licence, to which he at once acceded. We think, with due care and some improvements that have been suggested to him, this establishment will ultimately become a very useful one for the reception and treatment of incurables and idiotic patients.

We shall not unnecessarily trouble your Excellency by references to individual cases, which have been officially brought under our observation, and on which we have duly reported; or to lunatics confined singly in unlicensed houses, many instances of which we have become acquainted with during the past year, and in whose regard we have either personally or by letter communicated with their family or friends, for the purpose of bettering their condition, either by a change of residence, or by additonal means of support.

The returns of single lunatics under the Act 5 & 6 Vic., c. 123, sec. 36, are by no means regular. The law is easily evaded, so much so that unless the party in charge of the individual sends a voluntary intimation to the Inspector's office, we may say we can have none but accidental information on the subject. Patients, for example, are removed from asylums uncured, and we have not the means of tracing their subsequent abode, or even an authority to make inquiries thereon. Lunatics under the Lord Chancellor's protection are, no doubt, safe; but as to the others, it is impossible for us to say what treatment they ultimately receive. We are strongly inclined to think that all insane persons, whether placed on pension or otherwise, should be inspected from time to time, and individual reports made to the Lord Chancellor in each case.

---

